# Potential of the Desert Locust *Schistocerca gregaria* (Orthoptera: Acrididae) as an Unconventional Source of Dietary and Therapeutic Sterols

**DOI:** 10.1371/journal.pone.0127171

**Published:** 2015-05-13

**Authors:** Xavier Cheseto, Serge Philibert Kuate, David P. Tchouassi, Mary Ndung’u, Peter E. A. Teal, Baldwyn Torto

**Affiliations:** 1 Behavioral and Chemical Ecology Department, International Centre of Insect Physiology and Ecology (*icipe*), Nairobi, Kenya; 2 Chemistry Department, Jomo Kenyatta University of Agriculture and Technology, Nairobi, Kenya; 3 Chemistry Research Unit, Center for Medical, Agricultural, and Veterinary Entomology, United States Department of Agriculture/Agricultural Research Service, Gainesville, Florida, United States of America; CSIR- Indian Institute of Toxicology Research, INDIA

## Abstract

Insects are increasingly being recognized not only as a source of food to feed the ever growing world population but also as potential sources of new products and therapeutic agents, among which are sterols. In this study, we sought to profile sterols and their derivatives present in the desert locust, *Schistocerca gregaria*, focusing on those with potential importance as dietary and therapeutic components for humans. Using coupled gas chromatography-mass spectrometry (GC-MS), we analyzed and compared the quantities of sterols in the different sections of the gut and tissues of the locust. In the gut, we identified 34 sterols which showed a patchy distribution, but with the highest composition in the foregut (55%) followed by midgut (31%) and hindgut (14%). Fed *ad libitum* on wheat seedlings, five sterols unique to the insect were detected. These sterols were identified as 7-dehydrocholesterol, desmosterol, fucosterol, (3β, 5α) cholesta-8, 14, 24-trien-3-ol, 4, 4-dimethyl, and (3β, 20R) cholesta-5, 24-dien-3, 20-diol with the first three having known health benefits in humans. Incubation of the fore-, mid- and hindgut with cholesterol-[4-^13^C] yielded eight derivatives, three of these were detected in the gut of the desert locust after it had consumed the vegetative diet but were not detected in the diet. Our study shows that the desert locust ingests phytosterols from a vegetative diet and, amplifies and metabolizes them into derivatives with potential salutary benefits and we discuss our findings in this context.

## Introduction

The increasing world population has worsened the serious problem of food security especially in developing countries [1,2]. Under the new paradigm shift of “insects to feed the world”, insects are increasingly being recognized not only as a source of food to feed the ever growing world population but also as potential sources of new products and therapeutic agents [3–6]. In this regard, insects satisfy three important requirements: (i) they are an important source of protein and other nutrients; (ii) their use as food has ecological advantages over conventional meat and, in the long run, economic benefits for mass production as animal feed and human food, and (iii) a rich source of drugs for modern medicine [7, 8, 6, 9,10].

The desert locust, *Schistocerca gregaria* (Orthoptera: Acrididae), is an economically damaging pest of a wide range of crops, mainly cereals in the Sahel region of Africa [9]. It occurs in many parts of Africa and Asia. Major outbreaks have occurred in Eritrea, Somalia, Ethiopia, Djibouti, Yemen, Saudi Arabia and Madagascar [10]. Despite its alarming threat to food security, the locust is an excellent source of dietary components not only in many African resource poor communities [11], but also several other countries including; Thailand [12] and North East India [3,6].

To date, much of the research on the desert locust has focused on its control, physiology, nutrition, metabolism and biochemistry [13–17]. However, an emerging new dimension in the research on *S*. *gregaria* is on its potential as a source of compounds of dietary and therapeutic value for food and nutritional security in Africa. Nutrient analysis of the desert locust has shown that, about 62% of the dry weight of an adult desert locust consists of proteins, 17% as fats, with the remainder as inorganic constituents (Si, Cu, Fe, Mn, Na, K, Ca, Mg, Ti, Ni, P, S) [18,9]. Furthermore, the desert locust is easy to rear, requiring no special feeding mode. At the International Centre of Insect Physiology and Ecology, based in Nairobi, we have successfully mass reared a large gregarious colony of the insect on wheat seedlings for over a hundred generations, periodically infusing the colony with field samples to prevent genetic drift.

It is well established that dietary plant sterols lower plasma cholesterol concentrations by inhibiting intestinal cholesterol absorption [19,20]. Although vegetable oils and products based on them are generally the richest sources of phytosterols [21], insects can also be potential sources of useful sterols for humans. However, very little has been done in this context to the best of our knowledge.

Documentation of sterols in grasshoppers and the desert locust is narrowed to their use and metabolism by the same insects [19]. Like most arthropods, the desert locust is incapable of biosynthesizing sterols *de novo* from isoprenoid precursors, thus necessitating acquisition from dietary sources [13–17]. Sterols are well known to serve various roles in insects such as components of cellular membrane [13,15], precursors for several physiologically active metabolites and hormones like ecdysteroids, hedgehog signaling which controls cell proliferation and differentiation [22,23]. Given the paucity of data on the sterol composition of the desert locust, coupled with the importance as human food, the current study sought to 1) identify these compounds by gas chromatography-mass spectrometry and 2) profile potential therapeutic compounds present in the desert locust focusing on free sterols and their metabolites after feeding fifth instars of the insect on a plant-based diet.

## Materials and Methods

### Chemicals

Cholesterol (≥ 99%) was purchased from BDH chemicals (England), stigmasterol (~95%) and campesterol (~ 65% crystalline) were purchased from Sigma-Aldrich (California, USA). Lupeol, a compound isolated from *Fagara tessmanii*, was donated by Ivan Addae-Mensah (University of Ghana). Hydrox-sil was purchased from Chrompack (Holland). Cholesterol-[4-^13^C] (99 atom % 13 C) was purchased from CND-isotopes Pointe-Claire (Quebec, Canada).

### Plant material

Wheat (*Triticum aestivum* L.) seeds were purchased from the local market in Nairobi, grown in a screenhouse in 2 L plastic pots (17 cm diameter) filled with red soil, compost and sand (3:2:1, v/v/v) and were watered daily as previously described [24,25]. Fifty seeds were planted. The screenhouse was maintained at a temperature of 22 ± 1°C, 60–70% relative humidity (RH) and 12L: 12D photoperiod. Three weeks later, the seedlings were excised from the growing media and fed to fifth-instar nymphs of *S*. *gregaria*.

### Insects

Gregarious phase desert locusts were reared at the International Centre of Insect Physiology and Ecology (01°13’ 25.3” S, 36°53’ 49.2” E; ≈ 1,600 m ASL) at the Insect and Animal Rearing and Quarantine unit. Insects were reared on a diet of wheat seedlings and wheat bran in a room maintained at 30 ± 4°C, 40–50% RH and a photoperiod of 12:12 L:D. About 200–250 insects were reared in aluminum cages (50 x 50 x 50 cm) in a dedicated room (4.5 x 4.5 m), well aerated with a duct system to maintain a negative pressure [26].

### Extraction of sterols

The method used for the extraction of sterols was similar to that of [25], while the method previously described by [26] was used for partial purification and identification summarized as follows:

#### Extraction from wheat seedlings

Twenty-one day old fresh wheat seedlings were snap frozen in liquid nitrogen, crushed and 1 mg extracted in 1 ml pentane by sonication for 1 hr. The supernatant was pipetted into 1.1 ml crimp top tapered amber vials (Chromacol Ltd, UK), capped and centrifuged at 13,000 rpm for 5 min at 20°C. The supernatant was filtered, dried over anhydrous Na_2_SO_4,_ concentrated in a gentle stream of N_2_ to 100 μl. The filtrates were stored at -80°C until use. Three replicates were carried out with each replicate done on a different batch of wheat seedlings (one batch = one planting pot).

#### Extraction from tissue and secretions of *S*. *gregaria*

All extractions were performed using fifth-instar nymphs (2 d after moulting) of *S*. *gregaria*. For oral secretions, 200 μl was collected using microcapillary tube (Drummond Scientific Co. U.S.A) placed at the tip of the mouth of the locust as previously described [27]. The oral secretion was drained into the capillary by gravity with about 10 μl of secretion being obtained from one insect. Extracts of fore-, mid- hindgut and fat bodies were prepared by chilling the insects on ice and dissection under physiological saline (Infusion Medicare Ltd, Nairobi, Kenya).

The three gut sections and fat bodies from each individual were separately cut open and sliced to small pieces. Hemolymph extracts from both sexes were obtained by puncturing the abdomen with the sharpened tip of a 200 μl micropipette after which the fluid was allowed to flow into the capillary by gravity and then stored at -80°C. Approximately 40 μl of hemolymph was obtained per insect.

The hemolymph was transferred into a vial containing 70% methanol (500 μl), vortexed for one min and centrifuged at 13,000 rpm for 5 min at 5°C to remove precipitated protein. Fresh frass was collected from the rearing cage containing the stage of *S*. *gregaria*. The frass was snap frozen in liquid nitrogen, crushed to fine powder and 1 mg extracted in 1 ml of pentane.

Oral and hemolymph secretions were transferred into a 5 ml clear glass vial containing 200 μl of phosphate buffer (25 mM, pH 6.8), 1 ml of pentane and vortexed for 5 min. Individual extract for oral secretion (and all the other body extracts and tissues) was pipetted into 1.1 ml crimp top tapered amber vials (Chromacol Ltd, UK), capped and centrifuged at 13,000 rpm for 5 min at 20°C. The supernatant was filtered, dried over anhydrous Na_2_SO_4_ and concentrated to 100 μl in a gentle stream of N_2_. The extractions and analyses of oral secretion, foregut, midgut, hindgut, hemolymph were replicated three times for this study, with each replicate done on a different batch of insects (1batch = 1 rearing cage).

### Analysis of sterols

GC-MS in full scan mode was used to detect sterols in the extracts. For each sterol sample extracted, 100 μl was divided into two equal portions. The first portion was analyzed directly by GC-MS whereas the other was derivatized prior to analysis to aid in identification.

Serial dilutions of authentic standards of cholesterol, campesterol, stigmasterol and β-sitosterol (1-100pg/μl) were also analyzed by GC-MS in full scan mode to generate linear calibration curves (peak area vs. concentration) with the following equations; cholesterol [*y = 0*.*7694x + 3*.*6807* (R^2^ = 0.9991)], campesterol, [*y = 0*.*6995x + 2*.*8313* (R^2^ = 0.9735)], stigmasterol [*y = 0*.*4996x + 1*.*7696* (R^2^ = 0.9886)] and β-sitosterol [*y = 0*.*5999x + 2*.*3004* (R^2^ = 0.9327)] which served as the basis for the external quantification of these sterols. Internal standard was also used in this study to quantify other sterols. 1 μl of lupeol (200 pg/μl) was added to each of the prepared sample to allow quantification by comparison of areas of the underivatized and trimethylsilyl sterol derivatives. The limit of quantification (LOD) and limit of detection (LOD) of the method used in the analyses of the four authentic sterols was estimated at a signal-to-noise ratio of 3:1 and 10:1, respectively, by injecting a series of diluted standards with known concentrations. The LOD was determined to be 1.12, 1.06, 1.07 and 1.04 ng/μl and the LOQ to be 1.54, 1.22, 1.30 and 1.20 ng/μl for cholesterol, campesterol, stigmasterol and β-sitosterol, respectively.

#### Derivatization of sterols

The extracted sample was concentrated to dryness in a gentle stream of N_2_ to yield 0.5–5 mg. This was reacted with 1 ml Hydrox-sil in a 5 ml micro- reaction vial and then heated at 100°C for 1 h to give trimethylsilyl (TMSi) derivatives.

#### Instrument conditions

Sterols were analyzed by GC-MS on a 7890A gas chromatograph (Agilent Technologies, Inc., Santa Clara, CA, USA) linked to a 5975 C mass selective detector (Agilent Technologies, Inc., Santa Clara, CA, USA) by using the following conditions: inlet temperature 270°C, transfer line temperature of 280°C, and column oven temperature programmed from 35 to 285°C with the initial temperature maintained for 5 min then 10°C/min to 280°C held at this temperature for 10.5 min and finally 50°C/min to 285°C and held at this temperature for 29.9 min. The GC was fitted with a HP-5 MS low bleed capillary column (30 m × 0.25 mm i.d., 0.25 μm) (J&W, Folsom, CA, USA). Helium at a flow rate of 1.25 ml/min served as the carrier gas. The mass selective detector was maintained at ion source temperature of 230°C and a quadruple temperature of 180°C. Electron impact (EI) mass spectra were obtained at the acceleration energy of 70 eV. A 1.0 μl aliquot of extract was injected in the split/splitless mode using an auto sampler 7683 (Agilent Technologies, Inc., Beijing, China). Fragment ions were analyzed over 40–550 *m/z* mass range in the full scan mode. The filament delay time was set at 3.3 min for the underivatized samples and 8.6 min for the derivatized samples.

Cholesterol, campesterol, stigmasterol and β-sitosterol were identified by comparison of gas chromatographic retention time and fragmentation pattern with that of the authentic standards. When there was a lack of corresponding reference compounds, the structures were proposed on the basis of their general fragmentation and using reference spectra published by library–MS databases: National Institute of Standards and Technology (NIST) 05, 08, and additional support for identifications was obtained from mass spectral data of the trimethylsilyl derivatives of the sterols.

### Preparation of gut homogenates and enzyme assay

To prepare gut homogenates, five fifth-instar nymphs of *S*. *gregaria* were chilled in ice, dissected and washed under physiological saline. The foregut, midgut and hindgut were excised to yield 40 mg of each section. The tissues were then sliced and homogenized by vortexing for 5 min with 1 ml of phosphate buffer (25 mM, pH 6.8). The slurry obtained was considered as homogenate and was subsequently used for incubation experiments. The enzyme assay consisted of 225 μl of the respective gut homogenates and 25 μl of 10 mg ml^-1^ cholesterol or cholesterol-[4-^13^C] in water: saline (3:1, *v/v*) in a 1.5 ml Eppendorf tube for 12 h in a water bath at 35°C modified from [28]. The reaction was terminated by adding 500 μl of pentane which also served to extract the reaction products. The organic phase was dried over anhydrous Na_2_SO_4,_ concentrated in a gentle stream of N_2_ to dryness and then dissolved in 50 μl pentane and 1 μl analyzed by GC-MS.

### Statistical analysis

The peak areas of total ion chromatogram corresponding to sterols were obtained by GC-MS analysis and were used to compute concentrations either from the internal standard or from the calibration curve. Concentrations were expressed as mean ± standard error. Analysis of variance was carried out for each sterol found within wheat, oral secretion, gut, frass, hemolymph and fat bodies of the desert locust and means were separated using Tukey’s studentized HSD at 5% level of significance. R-statistical program 2.11.0 was used for data analyses [29]. We also compared the amounts of the sterols commonly detected in the wheat and the entire locust (i.e., total amounts in the tissues) in three replicate runs using a t-test at α = 0.05 level of significance.

## Results

**Fig 1:**
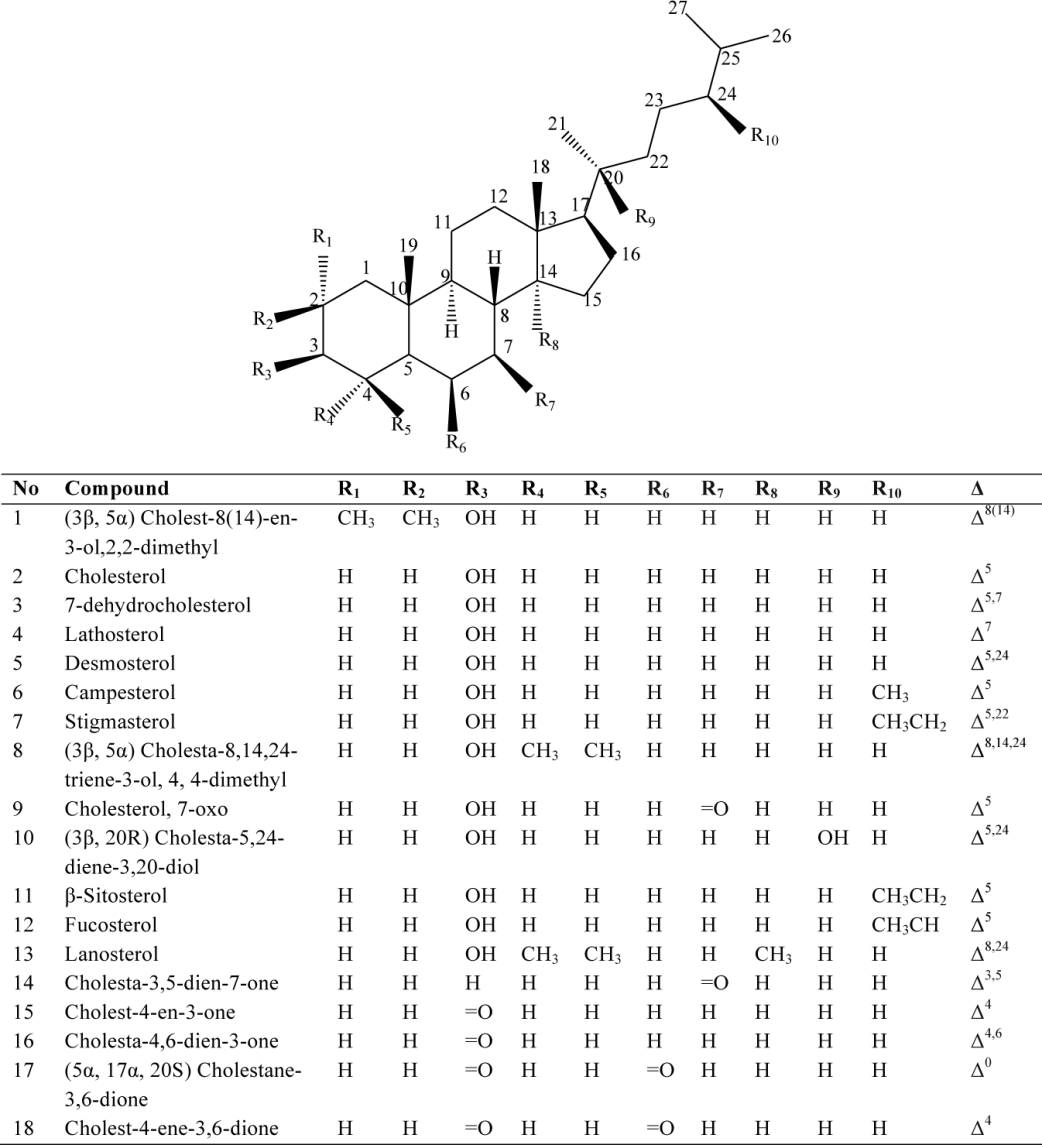
Structures of sterols. Δ indicates double bond and respective positions.

**Fig 2:**
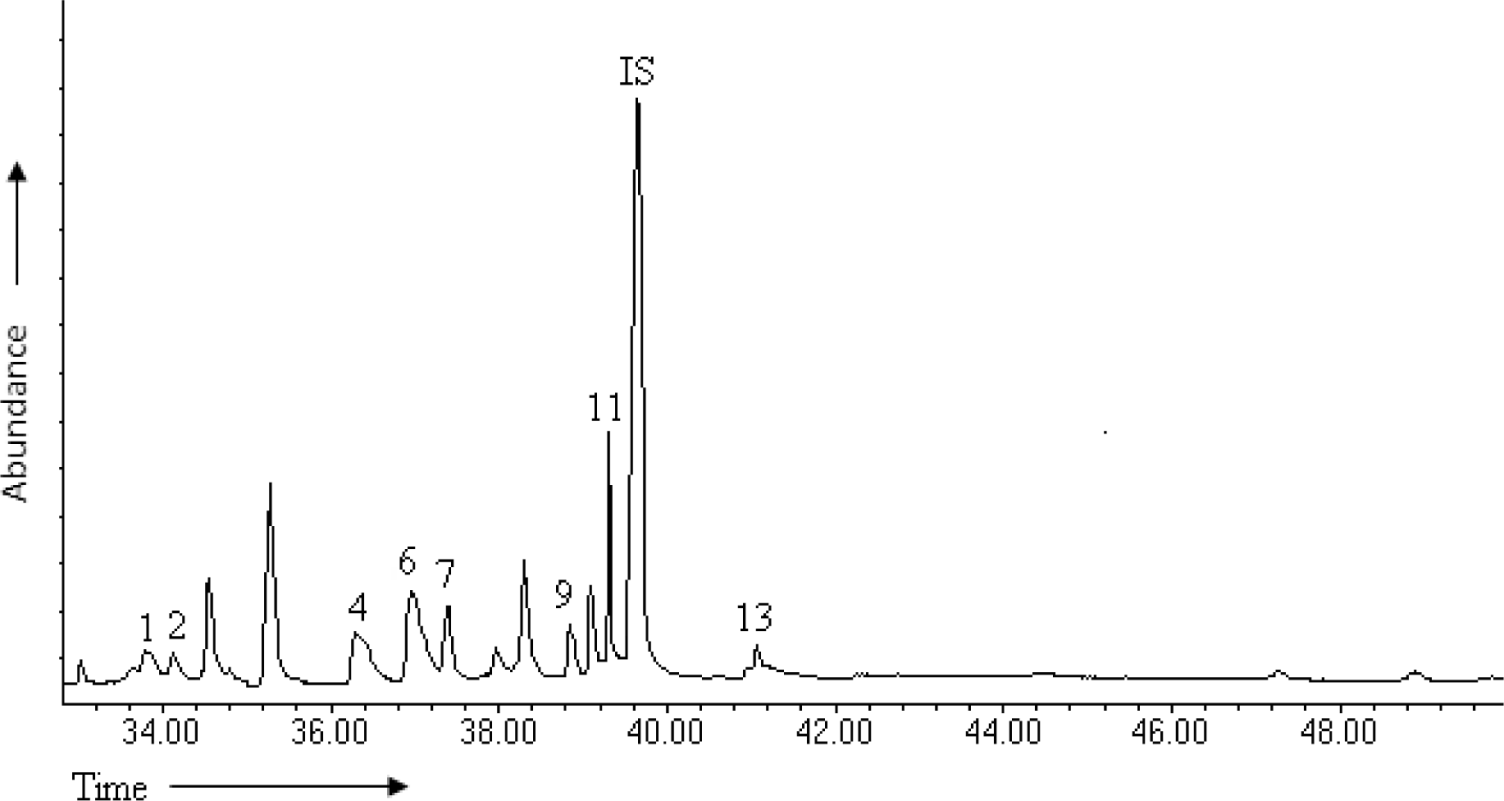
Total ion chromatogram of wheat seedlings extract. Numbers indicate eight of the thirteen sterols identified as shown in Table 1; IS, internal standard.

We identified eight different sterols in wheat seedling extract by GC-MS and confirmed identities of four using authentic standards for cholesterol, campesterol, stigmasterol and β-sitosterol. Cholest-8(14)-en-3-ol, 2,2-dimethyl (3*β*, 5*α*), lathosterol, cholesterol, 7-oxo and Lanosterol were tentatively identified based on mass spectral data and published library data (Figs 1 and 2).

Of these sterols, β-sitosterol [**11**] occurred in the highest concentration (119.92 pg/g) followed by campesterol [**6**] (103.54 pg/g) and stigmasterol [**7**] (95.06 pg/g) with the least being lanosterol [**13**] and cholesterol [**2**] (Fig 2, Table 1). Overall, the eight sterols detected in wheat seedlings were less abundant than their counterparts in other tissues or secretions (Table 1). A significantly higher amount of the sterols, cholest-8(14)-en-3-ol,2,2-dimethyl- (t = 23.7681, P<0.0001), cholest-5-en-3-ol (3 β) (t = 366.2564, P<0.0001), cholest-7-en-3-ol, (3β, 5α) (lathosterol), (t = 115.0105, P<0.0001), campesterol (t = 55.3092, P<0.0001), stigmasterol (t = 114.5298, P<0.0001), cholesterol, 7-oxo t = 50.0368, P<0.0001), β -sitosterol (t = 15.4871, P<0.0001), lanosterol (t = 30.1749, P<0.0001) were detected in the locust (i.e., total produced in all tissues) than in the wheat.

**Table 1:** Concentrations (pg/g) of sterols in 5^th^ instars desert locusts fed on wheat seedlings.

No	R_t_ (min)	Sterol	W	OS	FG	MG	HG	H	FB	F
1^◊^	34.00	Cholest-8(14)-en-3-ol, 2,2-dimethyl (3*β*, 5*α*)	14.19 ± 1.33 ^(a)^	87.08 ± 8.61 ^(a)^	1132.26 ± 64.60 ^(c)^	732.87 ± 15.04 ^(b)^	60.18 ± 1.53 ^(a)^	✘	✘	61.3 ± 1.86 ^(a)^
2 ^○^	35.45	Cholesterol	9.37 ± 0.93 ^(b)^	2865.43 ± 7.16 ^(e)^	9724.84 ± 24.59 ^(a)^	5931.60 ± 3.68 ^(d)^	88.81 ± 1.17 ^(b)^	1502.60 ± 4.18 ^(c)^	1880.47 ± 2.71 ^(f)^	88.26 ± 1.24 ^(b)^
3 ^◊^	35.85	7-Dehydrocholesterol	✘	463.19 ± 8.61 ^(d)^	6210.17 ± 12.56 ^(a)^	3812.89 ± 15.04 ^(c)^	✘	855.93 ± 2.78 ^(b)^	921.37 ± 4.40 ^(b)^	✘
4^◊^	36.02	Lathosterol	74.93 ± 2.23 ^(a)^	425.69 ± 13.04 ^(c)^	1527.47 ± 25.60 ^(d)^	912.87 ± 11.81 ^(b)^	73.94 ± 2.04 ^(a)^	✘	✘	73.73 ± 0.79^(a)^
5 ^◊^	36.94	Desmosterol	✘	57.38 ± 4.95 ^(d)^	8417.58 ± 30.04 ^(a)^	5146.68 ± 18.66 ^(c)^	✘	782.48 ± 2.08 ^(b)^	232.30 ± 2.71 ^(e)^	✘
6 ^○^	37.31	Campesterol	103.54 ± 2.04 ^(d)^	422.36 ± 32.01 ^(c)^	1711.04 ± 25.71 ^(e)^	1081.72 ± 8.01 ^(b)^	573.94 ± 2.05 ^(a)^	✘	✘	575.39 ± 1.88 ^(a)^
7^○^	37.40	Stigmasterol	95.06 ± 2.54 ^(a)^	601.26 ± 10.09 ^(c)^	1740.69 ± 16.38 ^(d)^	1087.6 ± 27.06 ^(b)^	88.6 ± 0.59 ^(a)^	✘	✘	88.72 ± 0.93 ^(a)^
8 ^◊^	38.90	(*3*β, *5α*) Cholesta-,14,24-trien-3-ol,4,4-dimethyl	✘	511.57 ± 10.05 ^(d)^	2882.16 ± 49.74 ^(a)^	1771.42 ± 18.77 ^(c)^	✘	364.58 ± 3.41 ^(b)^	102.88 ± 4.32 ^(e)^	✘
9 ^◊^	39.00	Cholesterol, 7-oxo	22.18 ± 1.09 ^(b)^	259.47 ± 5.75 ^(a)^	2825.98 ± 105.58 ^(d)^	1798.14 ± 19.74 ^(c)^	99.04 ± 0.89 ^(a,b)^	✘	✘	97.83 ± 1.25 ^(a,b)^
10 ^◊^	39.50	(3 β, 20R) Cholesta-5,24-dien-3,20-diol	✘	212.50 ± 7.16 ^(d)^	4530.97 ± 61.69 ^(a)^	2801.19 ± 22.59 ^(c)^	✘	745.86 ± 4.98 ^(b)^	1251.86 ± 2.90 ^(e)^	✘
11^○^	39.89	β -Sitosterol	119.92 ± 2.52 ^(b)^	556.41 ± 7.18 ^(d)^	758.60 ±77.65 ^(a)^	496.89 ± 15.42 ^(d)^	9.98 ± 1.44 ^(b)^	323.53 ± 7.41 ^(c)^	58.95 ± 2.41 ^(b)^	7.11 ± 0.65 ^(b)^
12 ^◊^	41.00	Fucosterol	✘	544.16 ± 4.28 ^(b)^	7571.51 ± 178.52 ^(a)^	4773.06 ± 30.41 ^(c)^	✘	519.05 ± 2.40 ^(b)^	436.55 ± 3.40 ^(b)^	✘
13 ^◊^	41.50	Lanosterol	10.07 ± 1.96 ^(c)^	1080.71 ± 2.83 ^(a)^	859.97 ± 128.41 ^(a)^	623.75 ± 11.60 ^(d)^	76.59± 12.23 ^(b,c)^	513.32 ± 6.12 ^(d)^	235.16 ± 1.90 ^(b)^	88.57 ± 1.51 ^(b,c)^

Retention time (R_t_), wheat seedlings (W), oral secretion (OS), foregut (FG), midgut (MG), hindgut (HG), frass (F), hemolymph (H) and fat body (FB).

Concentrations of sterol bearing the same letter in various tissues and secretions (across the column) are not significantly different (P≤0.05, Tukey’s, HSD test).

^○^ Identified by comparison with authentic samples.

^◊ ^Identification by library data and through derivatization.

✘ denotes not detected.

**Fig 3:**
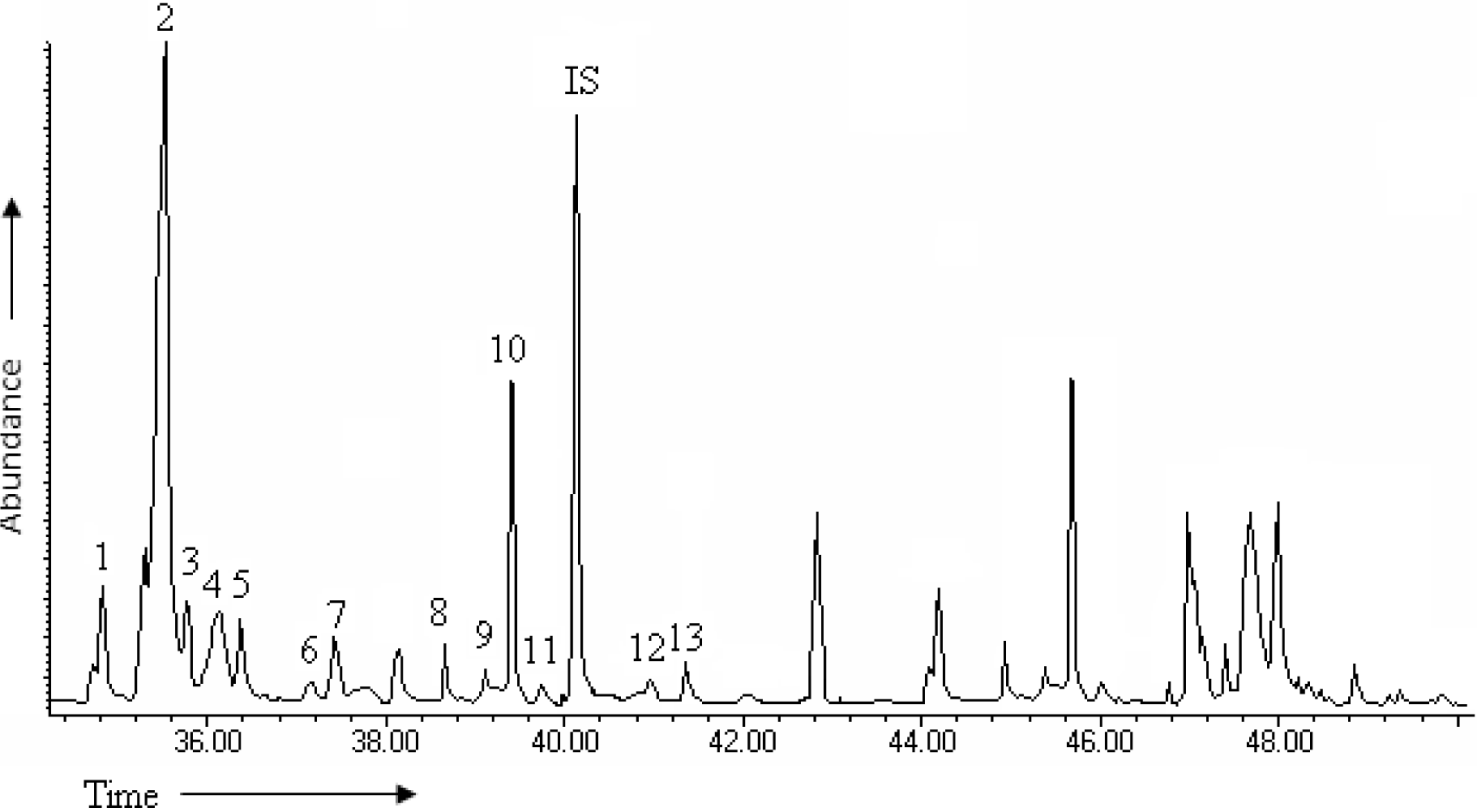
Representative total ion chromatogram of the oral secretion, foregut and midgut of the desert locust after feeding on wheat seedlings. Numbers 1–13 indicate sterols identified as shown in Table 1; IS, internal standard.

Of the thirteen sterols detected in the oral secretion, cholesterol [**2**] was the most abundant (2865 pg/g) while desmosterol [**5**] was the least abundant (Figs 1 and 3; Table 1).

The sterol composition of the oral secretion was similar to that of the fore- and midguts. The sterol profile in the frass was similar to that found for wheat seedlings and hindgut, with campesterol [**6**] being the most abundant sterol (575.39 pg/g), with cholesterol [**2**] and stigmasterol [**7**] detected in similar levels (Table 1).

Thirteen sterols were found in the fore- and the midgut tissues, eight of which were similar to those identified in the wheat seedling. Five new sterols detected in the oral secretion, fore- and midguts were tentatively identified as 7-dehydrocholesterol [**3**], desmosterol [**5**], (3β, 5α) cholesta-8, 14, 24-trien-3-ol, 4, 4-dimethyl [**8**], (3β, 20R) cholesta-5, 24-dien-3, 20-diol [**10**], and fucosterol [**12**] (Figs 1 and 3, Table 1). In both fore- and midgut tissues, cholesterol [**2**], demosterol [**5**] and fucosterol [**12**] were the most abundant while compounds **3**, **5**, **8**, **10**, and **12** were conspicuously absent in the hindgut and frass. Furthermore, eight sterols including the five which were not detected in the hindgut and frass were identified in the hemolymph and the fat body (Table 1).

### Enzymatic breakdown of cholesterol

When cholesterol and labeled cholesterol (cholesterol-[4-^13^C]) were separately incubated with various sections of the gut, eight different metabolites were detected in the fore- and midgut tissues but not in the hind gut (Table 2).

**Table 2:** Concentration (pg/mg) of cholesterol metabolites in various tissues after incubation with normal cholesterol (C) and labeled Cholesterol-[4-^13^C] (C-[4-^13^C]).

No	R_t_ (min)	cholesterol and metabolites	FG	MG	HG
2^○^	35.45	Cholesterol	290341.90 ± 2115.20	267450.70± 8828.00	298887.13±7925.00
3^◊^	35.76	7-Dehydrocholesterol	1193.79± 863.22	3487.80 ± 51.00	✘
4^◊^	35.94	Lathosterol	228.01± 18.85	2544.41± 51.35	✘
14^◊^	36.63	Cholesta-3,5-dien-7-one	107.17± 50.01	1624.89± 53.11	✘
15^◊^	37.40	Cholest-4-en-3-one	48.99± 23.12	593.40 ± 8.12	✘
16^◊^	37.98	Cholesta-4,6-dien-3-one	52.20± 9.87	1086.10 ± 26.77	✘
17^◊^	40.87	(5α,17α, 20S) Cholestan-3,6-dione	83.40± 9.89	826.17 ± 8.88	✘
18^◊^	41.11	Cholest-4-en-3,6-dione	68.09 ± 11.33	866.78± 5.25	✘
9^◊^	41.31	Cholesterol,7-oxo	26.08 ± 6.89	428.99± 6.86	✘

Retention time (R_t_), foregut (FG), midgut (MG) and hindgut (HG).

^○^ Identified by comparison with authentic samples.

^◊^ Identification by search in the spectra database and through derivatization.

✘ denotes not detected.

**Fig 4:**
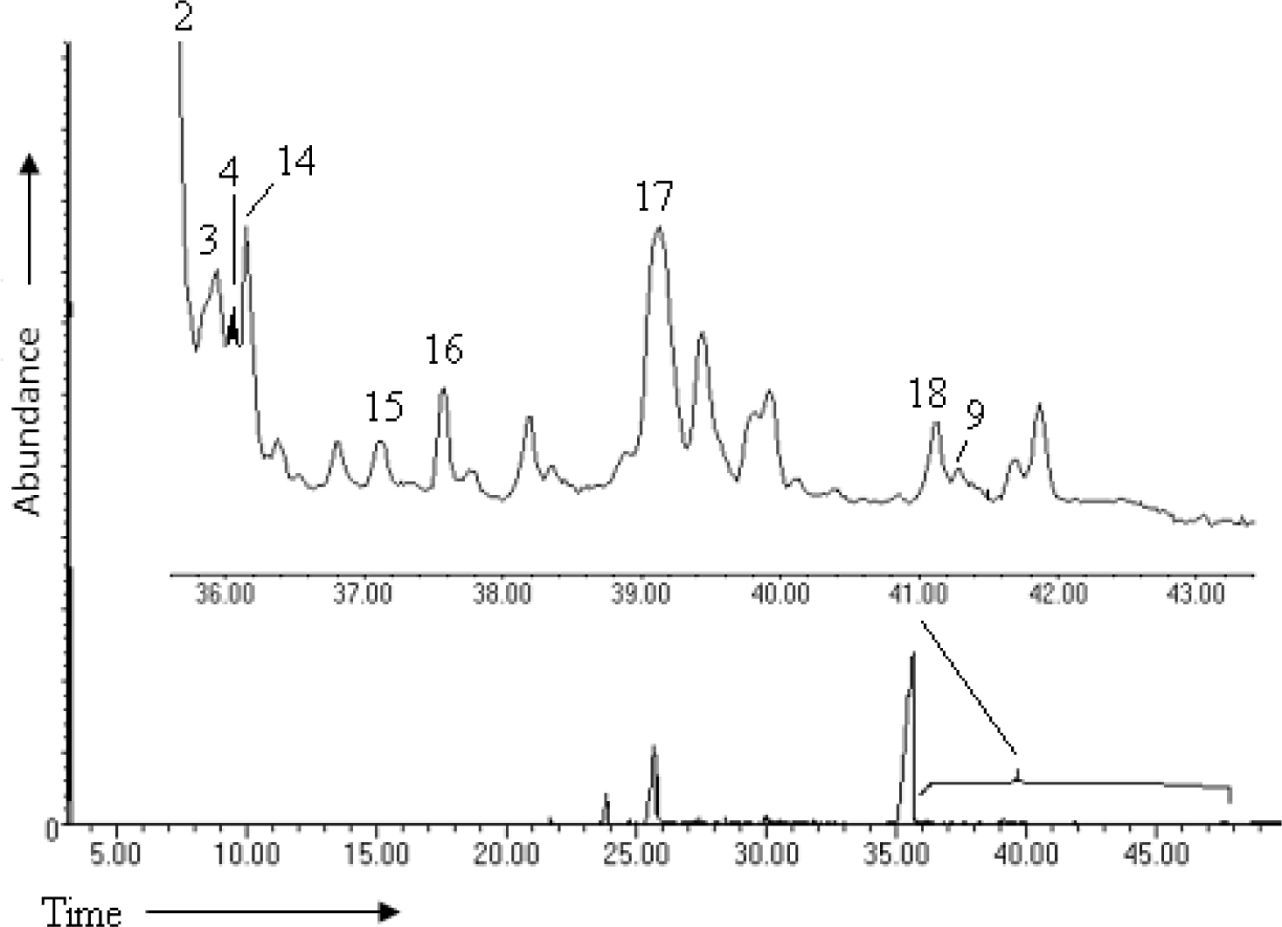
Representative total ion chromatogram of the foregut and midgut of the desert locust after incubation with cholesterol-[4-^13^C] with an expanded view of the identified cholesterol metabolites (retention time 35–50 min)].

The homogenates were dominated by cholesterol derivatives which were 3- to 20- fold more concentrated in the midgut than in the foregut. In both sections of the gut, 7-dehydrocholesterol (3487.8 pg/mg) [**3**] and lathosterol (2544.41 pg/mg) [**4**] were the most abundant metabolites; foregut 1193.79 pg/mg, 228.01 pg/mg, respectively (Figs 1 and 4, Table 2).

## Discussion

In this study we identified a significant number of sterols present in the desert locust and showed that different secretions and tissues contain both different sterols and ratios of sterols. Following ingestion of wheat seedlings by the desert locust, five sterols and unique to the insect were detected, and tentatively identified in the gut including 7-dehydrocholesterol **[3**], desmosterol [**5**], (3β, 5α) cholesta-8, 14, 24-trien-3-ol, 4, 4-dimethyl [**8**], (3β, 20R) cholesta-5, 24-dien-3, 20-diol [**10**], and fucosterol [**12**]. However, these metabolites were only present in the oral secretion, fore- and midgut tissues, and conspicuously absent in the hindgut and frass, suggesting their selective absorption across the insect tissues. Furthermore, the detection of these metabolites in the hemolymph and fat body extracts lends support for their absorption, transport by a suitable carrier and eventual storage and utilization.

The detection of these unique metabolites supports additional bioconversion that occurs in the insect possibly through enzymatic means or gut microbiota. Compounds **5, 8**, and **10** have been identified previously as dealkylated products of lanosterol while fucosterol is a metabolite of β-sitosterol [30, 31]. Therefore, the presence of lanosterol [**13**] metabolites in the oral secretion (OS), foregut (FG) and midgut (MG) suggests that the desert locust is likely to contain dealkylation enzymes or endosymbionts in the gut capable of performing this reaction [32].

The presence of 7-dehydrocholesterol, lathosterol and cholesterol, 7-oxo enzymatic products of cholesterol, and cholesterol in the oral secretions, fore-and midgut indicates that the metabolism of cholesterol starts early in the gut system. 7-Dehydrocholesterol is a metabolite of cholesterol and a key intermediate in the biosynthesis of ecdysone, initially shown to be formed through a direct bioconversion reaction restricted in the prothoracic gland [33]. An earlier study in *Drosophila pachea*, by [34] showed that it readily converted lathosterol [**4**] to 7-dehydrocholesterol [**3**]. By extension, our findings suggest that both the foregut and midgut of the desert locust are able to convert cholesterol to lathosterol [**4**], cholesterol 7-oxo [**9]** and 7-dehydrocholesterol [**3**]. As such, provided there is enough reason from our data to believe that cholesterol is indirectly converted to 7-dehydrocholesterol via either cholesterol 7-oxo or first to lathosterol before cholesterol 7-oxo, and eventually the latter is converted to 7,β-hydroxycholesterol and then 7-dehydrocholesterol.

Phytosterols have been shown to exert cardiovascular protective effects mainly via their cholesterol lowering ability, modulation of endothelial function and antioxidant capacity [35,36]. Other benefits include, anti-inflammatory [16], anticancer,[37] and immune regulatory effects[38]. Our study provides evidence that the desert locust has a rich composition of sterols comprising mostly phytosterols. Although cholesterol is the major tissue sterol as in other insects [39], our study shows that it is 1 out of a total of 13 sterols identified in the gut and other tissues of the desert locust. Phytosterols and cholesterol have a common target of absorption through the intestinal micelles in humans [18]. However, when both are consumed disproportionately, phytosterols are known to have a competitive advantage in terms of absorption over cholesterol as reported in a study using phytosterols to treat hypercholesterolemic patients [18]. Because of its high phytosterol content, our study suggests that, utilizing the desert locust as human food could be advantageous as it can help reduce high levels of serum cholesterol. (3β)-Cholesta-5,7-dien-3-ol also known as 7-dehydrocholesterol is a key ingredient in cutaneous synthesis of vitamin D in humans whose role ranges from maintaining healthy bones to fighting cancers, autoimmune diseases, infectious diseases, and cardiovascular diseases [37,38]. Therefore, consumption of the desert locust may provide these additional nutritional benefits.

All of the eight sterols identified in wheat seedlings were also detected in the desert locust and present in the oral secretion, foregut, midgut, hindgut and frass. Our findings show that fed *ad libitum* on wheat seedlings, the desert locust was able to ingest phytosterols from wheat seedlings. However, most of these common phytosterols appear to be amplified in the locust tissues as evidenced by the significantly higher amounts observed in the insect compared to wheat. This indicates that the locust can serve as a rich source for these compounds after it has fed on a vegetative diet. In wheat seedlings, β-sitosterol [**11**] occurred in the highest concentration followed by campesterol [**6**] and stigmasterol with the least being cholesterol [**2**]. This finding may reflect a general pattern of phytosterol abundance in plants in agreement with previous studies in flowering plants where β-sitosterol, campesterol and stigmasterol were identified as the major plant sterols whereas cholesterol if present was identified as a minor component [40,41].

In conclusion, results from our study show that the desert locust ingests phytosterols through a vegetative diet and, amplify and metabolize them into novel derivatives with potential human health benefits. Additionally, the locust has a rich nutritional composition in terms of proteins, fatty acids and minerals [9]. As such, the desert locust is a nutritious food source for both humans and animals.
